# Differences Between Human and Murine Tau at the N-terminal End

**DOI:** 10.3389/fnagi.2020.00011

**Published:** 2020-01-28

**Authors:** Félix Hernández, Jesús Merchán-Rubira, Laura Vallés-Saiz, Alberto Rodríguez-Matellán, Jesús Avila

**Affiliations:** ^1^Network Center for Biomedical Research in Neurodegenerative Diseases (CIBERNED), Carlos III Institute of Health, Madrid, Spain; ^2^Centro de Biología Molecular Severo Ochoa, Consejo Superior de Investigaciones Científicas (CSIC), Universidad Autonoma de Madrid (UAM), Madrid, Spain

**Keywords:** Alzheimer’s disease, human tau, murine tau, neurodegeneration, tauopathies

## Abstract

Human tauopathies, such as Alzheimer’s disease (AD), have been widely studied in transgenic mice overexpressing human tau in the brain. The longest brain isoforms of Tau in mice and humans show 89% amino acid identity; however, the expression of the isoforms of this protein in the adult brain of the two species differs. Tau 3R isoforms are not present in adult mice. In contrast, the adult human brain contains Tau 3R and also Tau 4R isoforms. In addition, the N-terminal sequence of Tau protein in mice and humans differs, a Tau peptide (residues 17–28) being present in the latter but absent in the former. Here we review the main published data on this N-terminal sequence that suggests that human and mouse Tau proteins interact with different endogenous proteins and also show distinct secretion patterns.

## Introduction

The microtubule-associated protein Tau is mainly expressed in neurons (Weingarten et al., 1975; Drubin and Kirschner, 1986). Tau promotes the assembly and stabilization of microtubules (for a review, see Avila et al., 2004). However, it has recently been suggested that rather than stabilizing the microtubules, Tau allows them to have labile domains (Baas and Qiang, 2019). In addition, and taking into account the subcellular localizations of Tau outside the axonal compartment, new functions have been proposed for this protein in dendrites (involvement in synaptic plasticity and regulation of NMDA/AMPA receptors), the nucleus (regulation of heterochromatin stability and integrity of cytoplasmic and nuclear RNA) and the membrane (interaction con F-actin and membrane proteins; for a review, see Sotiropoulos et al., 2017).

The hyperphosphorylation and aggregation of Tau in neurons is a common pathological hallmark of several neurodegenerative diseases known as tauopathies, Alzheimer’s disease (AD) being the most prevalent (Spillantini and Goedert, 2013). AD is characterized by the accumulation of extracellular plaques of β-amyloid peptide and intracellular neurofibrillary tangles (NFTs) formed by aggregated and hyperphosphorylated Tau protein. The amyloid cascade theory proposes that β-amyloid drives Tau phosphorylation and then Tau forms filaments and these filaments accumulate in NFTs (Selkoe and Hardy, 2016). The hypothesis suggests that β-amyloid accumulation precedes altered Tau metabolism. In addition, it has been demonstrated that a reduction of endogenous Tau ameliorates β-amyloid-induced deficits in AD (Rapoport et al., 2002; Santacruz et al., 2005; Roberson et al., 2007; Shipton et al., 2011). Under pathological conditions, the hyperphosphorylation of Tau protein prevents its binding to microtubules, resulting in its accumulation in the cytosol and consequent formation of intracellular NFTs. Aggregated Tau present in tauopathies does not seem to be the main toxic species. Instead, neuronal toxicity appears to be caused by smaller soluble aggregates or by specific conformations of Tau protein. In recent years, certain Tau conformations, called strains, have been linked to specific tauopathies (Sanders et al., 2014). Tau also accumulates in microglia and astrocytes in several of these conditions (Buée and Delacourte, 1999; Kovacs et al., 2016; Ferrer et al., 2018). The prion-like hypothesis proposes that Tau propagates from the entorhinal cortex and hippocampus to the cerebral cortex, thereby explaining in part the progression of AD (Braak and Braak, 1991). Tau is secreted and this extracellular form is taken up by neurons and glial cells (Gómez-Ramos et al., 2006; Calafate et al., 2015), thus contributing to the cell-to-cell spread of the protein (Holmes and Diamond, 2014; Medina and Avila, 2014).

Genetically altered mouse models have been widely used to study Tau metabolism (see https://www.alzforum.org/research-models) and have greatly contributed to our understanding of disease-related mechanisms. Furthermore, they are valuable for the evaluation of novel therapeutic approaches. Nevertheless, in addition to distinct splicing processes, mouse and human Tau protein show other differences. Here we focus on recent data suggesting that the N-terminal end of Tau protein explains why none of the murine models fully reproduces the complete spectrum of AD or related tauopathies.

## Differences Between Human and Murine Tau Gene

**Figure 1:**
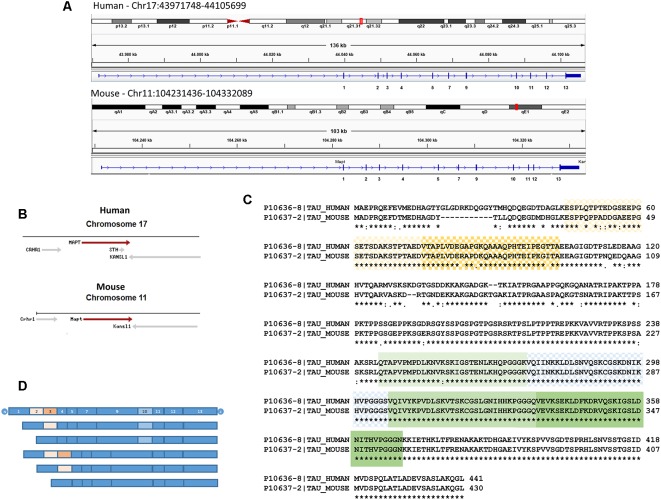
**(A)** Human and murine *tau* organization. Integrative Genomics viewer (using Human19 and Mouse mm10 genome versions) has been used to show the longest central nervous system (CNS) splicing isoforms (Tau 4R). Exons are shown by a vertical bar. Distances between exons are proportional to the sizes of the introns. Exons that undergo alternative splicing in the CNS: 2, 3 and 10 are indicated. It should be noted that although human and murine *tau* are similar size in the figure, the former covers 134 kb while the latter extends across 100 kb. The chromosomic localizations (red boxes) are also shown. **(B)** The genomic context for microtubule-associated protein tau gene (MAPT) in human chromosome 17 and mouse chromosome 11 are shown using data from The National Center for Biotechnology Information (NCBI; https://www.ncbi.nlm.nih.gov/). KANSL1, KAT8 regulatory NSL complex subunit 1; CRHC1, corticotropin-releasing hormone receptor 1; MAPT, Tau; STH, saitohin. **(C)** Sequence alignment of human Tau (entry UniProt number P10636-8) and mouse Tau (entry UniProt number sequence P10637-2) using the Clustal Omega program from the UniProt website. N-terminal domains, as well as microtubule-binding domains (MBD), are shown. In the figure, the same amino acids (*), while conservative amino acids (:) or less conservative ones (.) are highlighted. **(D)** A scheme of Tau isoforms present in the CNS. Six main transcripts are generated from a single gene. These isoforms are generated by alternative splicing of exons 2, 3, and 10. Exon 3 never appears independently of exon 2. Exons 1, 4, 5, 7, 9, 11, 12 and 13 are constitutive. Alternative splices of exons 2 (light orange), 3 (orange), and 10 (light blue) are shown in panels **(C,D)**.

Human *tau* (chromosome 17) spans 134 kb while murine *tau* (chromosome 11) stretches across 100 kb (for a review, see Poorkaj et al., 2001; Figure 1A). The genomic context for the microtubule-associated protein tau gene (MAPT) in humans and mice is similar (Figure 1B). MAPT through alternative splicing, give rise to distinct Tau isoforms in the central nervous system (CNS; Goedert et al., 1989; Andreadis et al., 1992). In the adult CNS, alternative splicing produces six distinct Tau isoforms, which differ in the presence or absence of exons 2, 3 and 10. While exon 2 can appear alone, exon 3 never appears independently of exon 2. Exon 10 encodes one of the four repeat sequences that form the microtubule-binding domain (MBD). Those isoforms that carry exon 10 result in Tau with four repeated microtubule-binding sequences (Tau 4R), while those without this exon have only three (Tau 3R; Figure 1D). The expression of these Tau isoforms is regulated by development. Tau 3R isoforms are present in early stages of development, while Tau 4R are found mainly in adults (for a review, see Avila et al., 2004; Wang and Mandelkow, 2016).

The main function of tau is to promote the polymerization of tubulins (Weingarten et al., 1975) and prevents their instability by its binding to microtubules (Drechsel et al., 1992). The Tau 4R isoform promotes microtubule assembly faster than Tau 3R (Goedert and Jakes, 1990). This observation suggests that the Tau 3R protein modulates brain development by decreasing the stability of microtubules. The expression of Tau 3R protein during development, as well as the absence of Tau 4R in the fetal human brain (Goedert et al., 1989; Kosik et al., 1989; Goedert and Jakes, 1990) support this notion. In the adult human brain, Tau 3R and Tau 4R isoforms are present in the same proportion (Avila et al., 2004). In addition to regulation through splicing, Tau phosphorylation is controlled during development, being higher in fetal neurons and decreasing with age (Brion et al., 1993). Tau phosphorylation during development correlates with the period of active neurite outgrowth, a process that requires a dynamic microtubule network.

The expression of Tau isoforms in the adult mice brain differs from humans. The Tau 3R isoform is not present in adult mice (Brion et al., 1993; Spillantini and Goedert, 1998). In this regard, adult hippocampal neurogenesis in mice is characterized by an unusual feature, namely Tau 3R is the main isoform present in newborn neurons in the hippocampal dentate gyrus (Bullmann et al., 2007) and in the subventricular zone (SVZ; Fuster-Matanzo et al., 2009). Doublecortin (DCX) is a microtubule-associated protein expressed in neural progenitor cells. DCX+ cells give rise to new neurons in the adult brain. DCX-positive neuroblasts express the Tau 3R isoform and Tau is also found in a phosphorylated form (Fuster-Matanzo et al., 2009). During the processes of differentiation to adult neurons, there is a progressive change towards Tau 4R in mature granule cells (Bullmann et al., 2007). These data support the notion that axonal outgrowth that takes place in these neuroblast DCX+ requires a dynamic microtubule network and that Tau protein contributes to this dynamic cytoskeleton. In this regard, neuronal polarity and axonal outgrowth take place during adult neurogenesis, microtubules express Tau isoforms with less affinity for microtubules as Tau 3R is hyperphosphorylated. Interestingly, it has been proposed that adult neurogenesis recapitulates neuronal development (Ming and Song, 2011) as Tau phosphorylation is higher in fetal neurons (Brion et al., 1993) and Tau 3R isoforms are found during early developmental stages (Avila et al., 2004).

In addition to the splicing differences between human and mouse Tau protein, intron 9 (between exon 9 and 10) of human *tau* has a region that can give rise to the expression of a protein known as saitohin (Conrad et al., 2002; Figure 1B). Saitohin is not present in mice. In this regard, although a homologous sequence is found in the mouse gene, the absence of an open reading frame in mice prevents Saitohin expression (Conrad et al., 2004).

## Differences Between Human and Murine Tau Protein

Tau of distinct origins shows some variability in primary sequence—variability in the N-terminal end being greatest (Nelson et al., 1996). Although mouse and human Tau sequences are similar, the latter contains 11 amino acids in the N-terminal end that are absent in mice (Figure 1C). These amino acids probably affect some functions in which Tau is involved. Tau adopts the so-called “paperclip” folding in solution, and the N- and C-terminal domains fold onto the microtubule-binding repeat domains (Carmel et al., 1996; Jicha et al., 1997). The presence of residues 17–28 in humans, making the N-terminal end longer than in mice, could have implications for the intramolecular interaction between the N- and C-terminal ends of the protein and the microtubule-binding repeat domains. Given the difference in the N-terminal sequence of human and mouse Tau, it can be speculated that the latter would be less likely to adopt this pathological “paperclip” conformation, something that has been already proposed (Ando et al., 2011).

Figure 1C shows that the domain around tyrosine-18 differs between the two species. Tyrosine-18 is phosphorylated by Src-family non-receptor tyrosine kinase Fyn (Lee et al., 1998, 2004) and Tyrosine-18 phosphorylation modulates NMDA receptor in primary neuronal culture (Miyamoto et al., 2017). Interestingly, Tau mediates the targeting of Fyn to postsynaptic dendrites (Ittner et al., 2010). At present, it is not clear whether tyrosine-18 phosphorylation is increased or decreased in human Tau compared with the murine form of the protein. Given that the NMDA receptor may be necessary for the toxic effect of the β-amyloid peptide (Ittner et al., 2010), differences in tyrosine-18 phosphorylation in mouse and human tau could be relevant. Of note, proline residues Pro213, Pro216, and Pro219, which are important for the binding of Tau protein to Fyn (Lau et al., 2016), are present in both human and murine Tau (see Figure 1).

The N-terminal projection region of Tau protein, which protrudes around 19 nm from the microtubule surface (Hirokawa et al., 1988), allows interaction with other proteins. Several N-terminal Tau interaction partners have been identified. The first interaction described was with the neural plasma membrane through its N-terminal projection domain (Brandt et al., 1995), thereby suggesting that Tau was a mediator of microtubule-plasma membrane interactions and thus plays an important role in neuritic development. Recently, a study using a heterologous yeast system has revealed that Tau interacts with Annexin A2 in a Ca2+-dependent manner *via* the N-terminal projection domain (Gauthier-Kemper et al., 2018). This observation, therefore, suggests that Tau links microtubules to the axonal plasma membrane through this domain. The same study described that Tau also interacts with Annexin A6, which localizes to the axon initial segment (AIS), where the binding of Tau may lead to its retention in the axonal compartment (Gauthier-Kemper et al., 2018).

Another approach to study the function of the N-terminal end of Tau protein, specifically the role of amino acids 18–28 present in the human sequence (Figure 1C), involved removing these amino acids from the full length recombinant human protein and then performing glutathione-S transferase (GST) pull-down assays and mass spectrometry analysis. Thus, it was found that the human-specific N-terminal Tau motif interacts with neuronal proteins such as Synapsin-1 and Synaptotagmin-1 (proteins involved in synaptic transmission), proteins of the 14–3–3 family, and Annexin A5 (Stefanoska et al., 2018).

To test for proteins that specifically and only bind to human Tau residues 16–26 (not present in murine Tau), a column containing this peptide linked to Sepharose was used to study human brain proteins that bound to the resin from control subjects and from patients with Alzheimer disease. Creatine kinase-B (CK-B), gamma-enolase and glyceraldehyde 3-phosphate dehydrogenase were observed to bind to this human Tau peptide. Interestingly, CK-B from brain extracts taken from patients with AD did not bind to Tau (Hernández et al., 2019). This observation could be attributable to the oxidation of CK-B in this disease (Hernández et al., 2019). CK-B is a brain isoform that phosphorylates creatine in the presence of ATP (Wallimann et al., 1992), and enolase and glyceraldehyde 3-phosphate dehydrogenase are critical enzymes in the glycolytic pathway. These proteins are related to energetic processes and can have a high impact on neuronal functions that require energy. Moreover, the brain is highly susceptible to oxidative imbalance, and changes in the level of ATP may induce neurodegeneration (Wang et al., 2014). The CK-B/phosphocreatine complex may supply the ATP required for axonal transport under conditions in which this energy supply is interrupted (Wallimann et al., 2011). In fact, creatine protects cortical axons from energy depletion *in vitro* (Shen and Goldberg, 2012). Regarding the other proteins, enolase moves along the axon (Brady and Lasek, 1981) and glyceraldehyde 3-phosphate dehydrogenase has been implicated in rapid axonal transport (Zala et al., 2013). These data suggest that the presence of this domain (residues 16–26) in human Tau supports energy supply by glycolysis in times of low oxygen levels and may explain, at least in part, the decrease in glucose metabolism observed in AD due to altered Tau metabolism (Butterfield and Halliwell, 2019). Given that these studies have been carried out with the peptide that is present in human but not murine Tau, it is reasonable to propose that either these interactions do not take place in the latter or that the interaction of these proteins with murine Tau differs.

## Differences Between Human and Murine Tau Secretion

Tau is mainly an intracellular protein, although it is also present in brain interstitial fluid (for a review, see Yamada, 2017). Tau protein is secreted *in vivo*, and this process appears to be regulated in physiological conditions and modulated by neuronal activity (Pooler et al., 2013). The exact mechanism of Tau release is unclear. However, several studies have demonstrated the extracellular presence of vesicle-bound and soluble free Tau (Saman et al., 2012; Kanmert et al., 2015; Wang et al., 2017). Although it is not clear how Tau can localize at the cell membrane, several reports demonstrate its presence in this structure (Brandt et al., 1995; Arrasate et al., 2000). This localization may indeed favor its further secretion. In addition, it should be noted that neuronal death can result in the release of Tau into the extracellular space, thereby contributing to extracellular Tau in pathological conditions.

The abnormal accumulation of intracellular Tau can be mediated through the cell-to-cell propagation of seeds of the protein. This observation has given rise to the hypothesis of a prion-like transmission to explain the propagation of the main neuropathological hallmarks of AD (Goedert et al., 2010). This hypothesis suggests that pathology begins in a part of the brain and spreads through this organ over time, for example from the entorhinal cortex (Braak and Braak, 1991) or/and from the locus ceruleus (Braak and Del Tredici, 2011). The nature of the Tau species involved in secretion, spreading and uptake, as well as the apparent selectivity that could explain why certain neurons are affected while others are not, remains unclear. Greater knowledge of this process will contribute to the development of new therapeutic approaches focused on stopping the spread of the disease and of new approaches to facilitate early diagnosis. Interestingly, human Tau lacking N-terminal amino acids 18–28 is less efficiently secreted than full-length Tau upon overexpression in Cos7 cells (Sayas et al., 2019). That study demonstrated that the sequence containing human Tau residues 18–28 acts as a binding motif for End Binding proteins and that this interaction facilitates Tau secretion to the extracellular space (Sayas et al., 2019). Although a comparative study with murine Tau protein that lacks this sequence at the N-terminal end has not been carried out, differences with the human protein would be expected. However, this does not imply that murine Tau is not secreted since the protein is also found in the extracellular compartment of wild-type mice (Yamada et al., 2011).

## Concluding Remarks

Given that decreased Tau expression is neuroprotective, strategies to achieve a reduction in its expression are among the most promising approaches for the development of AD therapeutic drugs (for a review, see Jadhav et al., 2019). In this regard, the evaluation of these strategies must first be validated in murine models. However, as shown in this mini-review, Tau metabolism in mice differs from that in humans, not only in the splicing process (presence of Tau 3R in adult humans but not in mice) but also in the primary sequence, mainly in the N-terminal end. These differences may limit the success of murine genetic models and explain why they do not fully reproduce the complete spectrum of AD pathology or related tauopathies.

## Author Contributions

All authors listed have made a substantial, direct and intellectual contribution to the work, and approved it for publication.

## Conflict of Interest

The authors declare that the research was conducted in the absence of any commercial or financial relationships that could be construed as a potential conflict of interest.
